# Comparison of the effect of sedation and general anesthesia on pattern and flash visual evoked potentials in normal dogs

**DOI:** 10.1186/s12917-022-03375-5

**Published:** 2022-07-13

**Authors:** Stephanie Chang, Danielle Zwueste, Barbara Ambros, Jonathan Norton, Marina L. Leis

**Affiliations:** 1 Department of Small Animal Clinical Sciences, Western College of Veterinary Medicine, University of Saskatchewan, 52 Campus Drive, Saskatoon, SK S7N 5B4 Canada; 2 Department of Surgery, College of Medicine, University of Saskatchewan, Saskatoon, SK S7N 5E5 Canada

**Keywords:** Pattern visual evoked potential, Flash visual evoked potential, Dexmedetomidine, Butorphanol, Propofol, Sevoflurane

## Abstract

**Background:**

Visual evoked potentials (VEPs) can provide objective functional assessment of the post-retinal visual pathway. This study compared the effects of sedation (butorphanol and dexmedetomidine) and general anesthesia (propofol and sevoflurane) on pattern and flash VEPs. Dogs (*n* = 13) underwent sedation or anesthesia and VEPs were obtained from 3 subcutaneous recording electrodes placed on the head (O1, Oz, O2).

**Results:**

Pattern VEPs could only be recorded under sedation and a maximum of 3 peaks were identified (N75, P100, N135). Flash VEPs could be recorded under both sedation and anesthesia and a maximum of 5 peaks were identified (N1, P1, N2, P2, N3). The latency of the N1 peak and the baseline-N1 amplitude were significantly longer under general anesthesia.

**Conclusion:**

Visual evoked potentials should be preferentially recorded in dogs sedated with dexmedetomidine and butorphanol, regardless of the stimulus.

**Supplementary Information:**

The online version contains supplementary material available at 10.1186/s12917-022-03375-5.

## Background

The clinical assessment of the visual pathway in companion animals is achieved through methods such as evaluating pupillary light reflexes, the menace response, maze testing under photopic and scotopic conditions, and a patient’s ability to track falling cotton balls. These methods can be subjective and inconsistent, however. Electroretinography (ERG) is a tool frequently used by veterinary ophthalmologists to quantify the electrical response of the outer retina and assess its function, and a variety of different protocols can be employed to differentiate between rod and cone function [1]. Similarly, visual evoked potentials (VEPs) may provide more reliable and objective diagnostic information regarding the function of the post-retinal visual pathway, as described in humans [2]. In other words, while the ERG’s output is the sum of outer retinal electrical activity, the VEP yields the sum of the electrical activity from the retina to the visual cortex.

Visual evoked potentials are representations of electroencephalographic activity of the visual cortex following light stimulation [2–4]. There are two main types of VEPs, depending on the stimulus used. Pattern VEPs (P-VEPs) are elicited by a reversing black and white checkerboard pattern, whereas flash VEPs (F-VEPs) are elicited by light flashes of very short duration [2]. The resultant potentials are recorded from electrodes on the scalp and they appear as waveforms of alternating positive and negative peaks. The latencies and amplitudes of these peaks can be quantified. Compared to the F-VEP, The P-VEP is most useful in assessing visual acuity and optic nerve function in both dogs and humans [5, 6]. However, the F-VEP is more practical for individuals with poor vision and who are poorly cooperative [2].

Visual evoked potentials have been used as a diagnostic aid in several human disorders including Alzheimer’s disease, glaucoma, and demyelinating disorders such as multiple sclerosis and have also been used for intraoperative monitoring during procedures such as pituitary tumor resection [6–9]. Efforts have been made to investigate VEPs in dogs, both in normal animals, dogs of advanced age, and canine cognitive dysfunction [10, 11]. Once this diagnostic modality becomes more widely available and utilized in veterinary medicine, it may aid in early recognition, response to therapy, and prognostication of diseases such as glaucoma and demyelinating disorders.

Practically speaking, the P-VEP is more challenging to perform in veterinary patients as it requires visual fixation and this is most commonly achieved under general anesthesia [12]. There remain, however, several unanswered questions that must be addressed before VEPs can be reliably used in a veterinary clinical setting. The need to obtain multiple recordings and poor patient compliance often requires that VEPs in veterinary patients be recorded under sedation or anesthesia, although CNS depressants may alter VEP parameters [3]. Only a few studies have investigated the effects of anesthetic agents such as sevoflurane and there remains a lack of direct comparison between the effect of sedation and general anesthesia protocols on VEPs [3]. There is also limited data on the specific differences between pattern and flash stimulation on VEP waveforms in companion animals.

The purpose of this study was to determine normative latency and amplitude values for pattern and flash VEPs under a commonly used sedation and general anesthesia protocol, as well as to directly compare the effects of sedation and anesthesia on P-VEP and F-VEP waveforms, in clinically normal dogs.

## Results

### Physical, neurological, and ophthalmic examinations

The head and neck, integumentary, and musculoskeletal examinations were within normal limits. Thoracic auscultation and abdominal and peripheral lymph node palpation were unremarkable. All findings of the neurological examination were within normal limits and included evaluation of mentation, posture and gait, cranial nerves, postural reactions, spinal reflexes, and pain perception. Retinoscopy revealed the median refractive power for all dogs was − 1.5 D (range − 2 to 0.5 D). Electroretinography for all dogs was within normal limits and revealed a mean a-wave implicit time of 14.9 ms (range 5.9 to 20 ms), a mean b-wave implicit time of 35.2 ms (range 30 to 41.6 ms), a mean a-wave amplitude of 26.5 uV (range 4.5 to 81.7 uV) and a mean b-wave amplitude of 173 uV (range 104.2 to 304.8 uV).

### Pattern-VEPs

**Fig. 1:**
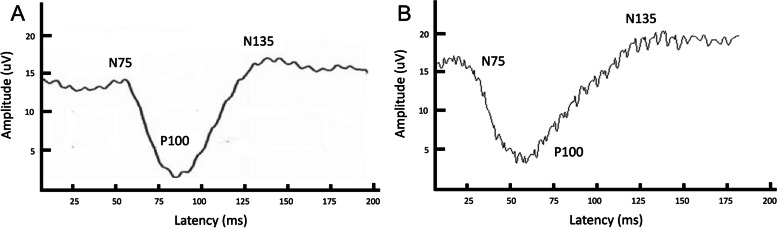
A typical P-VEP waveform obtained under sedation with butorphanol and dexmedetomidine (**A**) and general anesthesia with propofol and sevoflurane (**B**), recorded from the Oz electrode

Peaks were labeled as negative (N) 75, positive (P) 100 and N135 in accordance with previously published guidelines (Fig. 1) [2]. Pattern VEPs were reliably recorded in all dogs under sedation, with a maximum of 3 peaks identified. Pattern VEPs could not be recorded reliably under general anesthesia, as there was no peak that could be consistently identified in all 13 dogs. Peak latencies and amplitudes are reported in Tables 1 and 2. Statistical analysis of amplitudes and latencies with sedation versus general anesthesia could not be performed due to an insufficient number of recorded peaks. Sex did not significantly affect any of the measured latencies or amplitudes for Pattern VEPs (*P* > 0.05).

**Table 1:** Pattern visual evoked potential (P-VEP) peak latencies

	Sedation			General Anesthesia		
	O1	Oz	O2	O1	Oz	O2
N75						
Latency (ms)	48.46 (33.82–57.97)	48.36 (24.46–57.67)	48.54 (29.89–55.82)	43.78 (35.33–117.94)	46.44 (43.48–118.45)	46.88 (43.18–119.46)
n(%)	13 (100%)	13 (100%)	13 (100%)	7 (53.8%)	7 (53.8%)	7 (53.8%)
P100						
Latency (ms)	81.30 (70.2–89.75)	82.82 (73.22–91.03)	82.42 (73.30–89.98)	80.53 (69.70–173.89)	81.69 (69.91–173.39)	129.68 (99.12–205.14)
n(%)	13 (100%)	13 (100%)	13 (100%)	6 (46.2%)	6 (46.2%)	6 (46.2%)
N135						
Latency (ms)	132.06 (125.30–162.74)	134.89 (125.45–158.21)	135.91 (115.04–158.15)	46.88 (43.18–119.46)	83.18 (67.94–172.88)	130.59 (99.97–203.63)
n(%)	8 (61.5%)	8 (61.5%)	8 (61.5%)	7 (53.8%)	6 (46.2%)	6 (46.2%)

Latency is presented as median (range)

n (%) – number of dogs (percent of total) exhibiting that peak

**Table 2:** Pattern visual evoked potential (P-VEP) peak amplitudes

	Sedation			General Anesthesia		
	O1	Oz	O2	O1	Oz	O2
Baseline-N75						
Amplitude (uV)	0.94 (0.37–3.88)	0.82 (0.35–4.42)	0.90 (0.26–3.82)	2.6 (1.40–3.03)	2.49 (1.07–3.61)	1.36 (0.94–3.68)
n (%)	13 (100%)	13 (100%)	13 (100%)	7 (53.8%)	7 (53.8%)	7 (53.8%)
N75-P100						
Amplitude (uV)	8.49 (2.98–3.88)	10.82 (3.88–23.45)	8.95 (4.00–21.07)	2.95 (0.89–3.73)	2.55 (0.75–4.09)	2.90 (0.76–4.21)
n (%)	13 (100%)	13 (100%)	13 (100%)	6 (46.2%)	6 (46.2%)	6 (46.2%)
P100-N135						
Amplitude (uV)	7.97 (2.98–19.78)	10.17 (6.33–15.94)	8.96 (5.69–24.34)	1.70 (0.57–4.59)	1.69 (0.67–3.93)	1.43 (0.56–3.57)
n (%)	8 (61.5%)	8 (61.5%)	8 (61.5%)	6 (46.2%)	6 (46.2%)	6 (46.2%)

Amplitude is presented as median (range)

n (%) – number of dogs (percent of total) for which peak amplitude could be measured

### Flash-VEPs

**Fig. 2:**
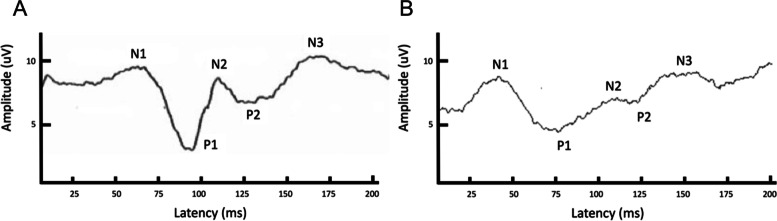
A typical F-VEP waveform obtained under sedation with butorphanol and dexmedetomidine (**A**) and general anesthesia with propofol and sevoflurane (**B**), recorded from the Oz electrode

Peaks were labeled as negative (N) or positive (P) in numerical sequence according to previously published guidelines (Fig. 2) [2]. Flash VEPs were reliably recorded in all dogs under sedation, with a maximum of 5 peaks identified. Flash VEPs could also be reliably recorded in animals under general anesthesia with N1 identified in all animals. Peak latencies and amplitudes are reported in Tables 3 and 4. Statistical analysis was only performed for the N1 latency and baseline to N1 amplitude as the N1 peak was most consistently observed under both sedation and general anesthesia. For all three recording electrodes, the amplitudes and latencies were significantly greater under general anesthesia than under sedation (Tables 3 and 4). The treatment order, sedation or general anesthesia first, did not affect N1 latency or baseline-N1 amplitude for any recording electrode (*P* > 0.05). Sex did not significantly affect any of the measured latencies or amplitudes for Flash VEPs (*P* > 0.05).

**Table 3:** Flash visual evoked potential (F-VEP) peak latencies

	Sedation			General Anesthesia		
	O1	Oz	O2	O1	Oz	O2
N1						
Latency (ms)	59.95^*^ (49.90–78.48)	60.84^**^ (50.27–74.35)	61.59^***^ (49.37–72.16)	66.81 (47.28–79.94)	66.80 (46.60–80.01)	66.96 (46.45–78.50)
n (%)	13 (100%)	13 (100%)	13 (100%)	13 (100%)	13 (100%)	13 (100%)
P1						
Latency (ms)	90.81 (79.71–102.43)	89.60 (78.88–104.24)	90.05 (79.64–105.00)	111.87 (75.18–136.47)	110.25 (76.39–133.45)	111.12 (77.29–133.76)
n (%)	13 (100%)	13 (100%)	13 (100%)	10 (76.9%)	9 (69.2%)	9 (69.2%)
N2						
Latency (ms)	115.57 (84.39–131.11)	115.64 (85.15–134.82)	112.24 (84.24–133.08)	129.98 (91.79–156.10)	139.95 (91.49–151.27)	139.19 (92.39–155.38)
n (%)	13 (100%)	13 (100%)	13 (100%)	9 (69.2%)	9 (69.2%)	9 (69.2%)
P2						
Latency (ms)	151.35 (127.75–171.20)	153.01 (128.73–174.59)	154.51 (128.25–173.39)	165.16 (140.5–186.90)	165.31 (142.39–187.05)	166.67 (142.69–187.95)
n (%)	13 (100%)	13 (100%)	13 (100%)	7 (53.8%)	6 (46.2%)	7 (53.8%)
N3						
Latency (ms)	182.67 (158.25–203.35)	185.99 (158.47–174.59)	185.92 (158.24–205.32)	185.69 (175.27–218.00)	188.89 (170.29–222.83)	186.31 (175.72–216.79)
n (%)	13 (100%)	13 (100%)	13 (100%)	7 (53.8%)	6 (46.2%)	6 (46.2%)

Latency is presented as median (range)

n (%) – number of dogs (percent of total) exhibiting that peak

^*^Significantly different (*P* = 0.045) latency compared to under general anesthesia

^**^Significantly different (*P* = 0.03) latency compared to under general anesthesia

^***^Significantly different (*P* = 0.044) latency compared to under general anesthesia

**Table 4:** Flash visual evoked potential (F-VEP) peak amplitudes

	Sedation			General Anesthesia		
	O1	Oz	O2	O1	Oz	O2
Baseline-N1						
Amplitude (uV)	4.55^*^ (1.42–11.56)	4.30^**^ (1.47–10.57)	4.39^***^ (1.38–11.10)	8.46–7 (5.96–12.40)	8.66 (4.58–15.77)	8.45 (5.71–13.57)
n (%)	13 (100%)	13 (100%)	13 (100%)	13 (100%)	13 (100%)	13 (100%)
N1-P1						
Amplitude (uV)	4.16 (1.63–10.93)	3.81 (1.78–11.64)	4.50 (1.68–10.85)	4.02 (0.18–16.71)	3.92 (0.25–17.07)	3.57 (0.29–16.37)
n (%)	13 (100%)	13 (100%)	13 (100%)	10 (76.9%)	9 (69.2%)	9 (69.2%)
P1-N2						
Amplitude (uV)	1.93 (0.69–11.00)	2.18 (0.37–11.64)	2.26 (0.70–11.17)	0.71 (0.24–1.68)	0.56 (0.19–1.24)	0.51 (0.34–0.88)
n (%)	13 (100%)	13 (100%)	13 (100%)	10 (76.9%)	9 (69.2%)	9 (69.2%)
N2-P2						
Amplitude (uV)	5.11 (0.95–11.37)	4.95 (1.31–12.89)	4.85 (1.29–12.93)	3.50 (0.25–5.71)	3.05 (0.89–5.87)	2.52 (0.57–6.3)
n (%)	13 (100%)	13 (100%)	13 (100%)	6 (46.2%)	5 (38.5%)	7 (53.8%)
P1-N3						
Amplitude (uV)	1.78 (0.75–9.31)	2.12 (0.99–10.22)	1.64 (0.80–9.65)	0.83 (0.40–4.96)	0.97 (0.13–4.69)	0.91 (0.24–5.36)
n (%)	13 (100%)	13 (100%)	13 (100%)	7 (53.8%)	6 (46.2%)	7 (53.8%)

Amplitude is presented as median (range)

n (%) – number of dogs (percent of total) for which amplitude could be measured

^*^Significantly different (*P* = 0.001) amplitude compared to under general anesthesia

^**^Significantly different (*P* = 0.003) amplitude compared to under general anesthesia

^***^Significantly different (*P* = 0.015) amplitude compared to under general anesthesia

## Discussion

Pattern VEPs could not be reliably recorded under general anesthesia in the present study, with each peak being undetectable in approximately 50% of the dogs. In order for P-VEPs to be successfully recorded, the dog must have the visual acuity to resolve the individual checks [5]. A previous investigation found that when the check size, or visual angle, was a minimum of 56 arc-min the P100 peak could be identified in 100% of tested dogs [5]. In our study the visual angle was 60 arc-min and the N75 and P100 peaks were identified in all dogs under sedation, suggesting that visual acuity does not account for this difference. Rather the loss of P-VEP appears to be due to the effects of general anesthesia.

Pattern VEP recordings have been previously successful in dogs induced and anesthetized with sevoflourane [3]. Anesthesia was induced in these dogs with sevoflurane delivered by mask. In clinical settings it is more common to induce anesthesia with an injectable agent such as propofol as it improves both speed and quality of the induction [13]. The dogs in the current study were thus first induced by propofol and then maintained under general anesthesia with sevoflurane. In addition to frequent clinical use, propofol is the anesthetic agent of choice for intra-operative monitoring of VEPs in humans and has the lowest suppressive effect of all intravenous anesthetics [14–16]. The median dose in the current study was 6.0 mg/kg with a maximum of 12 mg/kg. It has been suggested that large doses or boluses of propofol may still suppress, if not result in the complete loss of, VEPs [14, 17]. Similarly, it has been reported that anesthesia and sedation significantly attenuated and delayed waveforms in Beagles receiving electroretinography [18]. The suppressive effect of general anesthesia may account for the lack of successful VEP recordings in our study.

The use of dexmedetomidine and butorphanol allowed for successful detection of P-VEPs, with the N75 and P100 peaks observed in all dogs. This common combination of sedatives has not previously been used when evaluating VEPs in canines and the results of this study add to normative latency and amplitude data available to veterinarians. Dexmedetomidine similarly allowed for recording of VEPs in 74% of human cases with no impact on latency or amplitude [19]. Pattern VEPs have been evaluated in dogs using medetomidine, a less potent α-2 agonist than dexmedetomidine, in combination with midazolam and butorphanol [5]. Dexmedetomidine can be used as a premedicant and when given intravenously to dogs at a dose of 2 μg/kg allowed for significantly lower doses of propofol (2.7 ± 0.5 mg/kg) as an induction agent [20]. It is possible that pretreating dogs with dexmedetomidine and butorphanol will improve P-VEP recordings in dogs under general anesthesia due to this anesthetic sparing property. Given the success of P-VEP recording under sedation, however, using dexmedetomidine/butorphanol alone may provide a more practical clinical protocol and make the VEPs more available and attractive to both clinicians and clients.

Unlike the P-VEPs, F-VEPs were successfully recorded under both sedation and general anesthesia. Sedation allowed all 5 peaks to be recorded in all dogs. Under anesthesia the N1 peak was recorded in all animals with the following two peaks, P1 and N1, recorded in approximately 70% of animals. The P2 and N3, peaks, however, were only present in approximately 50% of the dogs. The reason for the increased frequency of the early peaks can be attributed to the anatomical basis of each peak. The visual pathway between the retina and the brainstem generates the first three peaks [21]. This portion of the pathway contains the optic nerve and optic tract and relies heavily upon axonal conduction [17]. The final two peaks correlate to the aspect of the visual pathway between the brainstem and cortex, which is much more heavily dependent on synaptic transmission [22]. General anesthesia inhibits neurotransmission and would consequently be expected to have a greater effect on polysynaptic portions of the visual pathway and therefore depress the last 2 F-VEP peaks the most [17].

The latency for N1 and amplitude for baseline-N1were significantly larger under general anesthesia compared with sedation for F-VEPs. This is in contrast to most studies, in which general anesthesia typically prolongs latency and actually reduces amplitude [17, 20, 23]. These studies have been in humans, however, and have compared awake patients with those under anesthesia. This is the first study to directly compare the effects of sedation versus general anesthesia on VEPs in dogs. There was likely increased suppression of the underlying EEG with general anesthesia compared to the sedation protocol, allowing for improvement of the signal:noise ratio and amplitude detection [13]. EEG amplitudes were shown to increase in humans anesthetized with 0.9% isoflurane, another volatile anesthetic, but then decrease again with 1.2 and 1.5% isoflurane anesthesia [24]. Unfortunately this cannot be confirmed as EEGs were not concurrently recorded in this study. Overall, the latencies were longer and amplitudes lower than those recorded from F-VEPs during which dogs were manually restrained only [4]. It remains unclear as to why P-VEPs are more affected by general anesthesia and further studies are warranted.

Existing investigations into VEPs in dogs have focused on recording from only the Oz location [3, 4, 10, 11]. This study, however, also investigated recordings from the more lateral electrode positions of O1 and O2. Recordings were successfully obtained from all three electrodes under sedation for both F-VEPs and P-VEPs and under general anesthesia for F-VEPs. The latency and amplitudes of each peak were similar across recording electrodes. The benefit of recording from laterally placed electrodes is that it provides a more accurate assessment of the visual pathway at and caudal to the optic chiasm [2, 5]. Dysfunction of these more caudal parts of the pathway will lead to asymmetry of the VEPs which can only be detected with a minimum of 2 recording electrodes [2]. Such asymmetries include differences in peak distribution, amplitude and polarity [25]. Now that normative data have been established for all three recording positions, clinicians will be better able to assess the functional effects of disease processes such as neoplasia, storage disorders and inflammatory diseases, all of which are known to occur in the region of the post-chiasmal visual pathway [26].

One limitation of this study is that refractive power of the dogs was not corrected. Refractive power represents the ability of the eye to focus light on the retina and deviations of this power can lead to blurred vision, which has the potential to affect P-VEP results [5, 27]. It was shown in a previous study that the most stable P-VEPs could be recorded with a refractive power of − 2.0 D [27]. Although refractive power was not corrected for in the present study, the median value of − 1.5D was close to the reported ideal of − 2.0D. While statistical differences in latencies and amplitudes have been documented between − 2 D and other refractive powers, the difference between -2D and − 1.5 D has not been investigated. The effect of this 0.5 D difference is thus unknown but is likely to be minimal. Another limitation is that the low frequency filter was set to 1 Hz in accordance with current clinical standards [2]. It has recently been demonstrated that the reproducibility of VEP recording under general anesthesia can be improved by increasing this filter to 10 Hz [13]. We may have been able to improve both our flash and pattern VEP results by making such an adjustment.

## Conclusion

Flash VEPs could be recorded under commonly used clinical sedation (dexmedetomidine and butorphanol) and general anesthesia (propofol and sevoflurane) protocols in dogs, although peaks were more consistently identified under sedation and both amplitude and latency were increased by anesthesia. Pattern VEPs could only be consistently recorded under sedation. Visual evoked potentials should be preferentially recorded in dogs sedated with dexmedetomidine and butorphanol, regardless of the stimulus.

Now that it has been demonstrated that both P-VEPs and F-VEPs can be successfully recorded using clinically relevant sedation protocols, future investigations should focus on the application to clinical cases. Detection of the loss of peaks and variances in amplitude and latency may allow the clinician to diagnose visual deficits earlier than current physical examination techniques. Documenting changes to the VEP over time may allow for the objective monitoring of a patient’s response to treatment and early detection of improvement in visual function.

## Material and methods

Thirteen dogs were used in this study. All dogs were adult beagle crosses, 6 of which were spayed females and 7 of which were castrated males. All dogs were between 3 and 4 years of age and weighed 9.2–22.9 kg (mean 16.6 kg). The length and width of each head was measured from the medial canthus to the external occipital protuberance and from the dorsal base of each pinna, respectively. The heads were 12.0–13.8 cm (mean 12.8 cm) in length and 10.0–14.0 cm (mean 13.0 cm) in width. All dogs received a complete physical, neurological and ophthalmic examination (including slitlamp biomicroscopy (Kowa SL-17 Portable Slit Lamp, Kowa Co, Tokyo, Japan), indirect ophthalmoscopy (Heine Omega 500, Heine Instruments Canada, Kitchener, Canada), and flash electroretinography (Sierra Sumitt, Cadwell, WA USA) prior to enrollment. All eyes were dilated using 0.5% tropicamide (Mydriacyl, Alcon Canada, Mississauga, Ontario) for posterior segment examination and electroretinography. For flash electroretinography, Proparacaine 0.5% ophthalmic solution (Alcaine, Novartis Pharma Canada, Dorval QC) was applied to the ocular surface to place the active electrode, a corneoconjunctival contact lens (ERG-jet; Universo SA, La Chaux-de-Fonds, Switzerland). Two needle electrodes (Cadwell low profile needle electrodes; Cadwell Laboratories, Kennewick, WA, USA) were positioned subcutaneously; the ground electrode was placed over the sagittal crest and the reference electrode was placed approximately 1 cm posterior to the lateral canthus. Using a handheld mini-Ganzfeld, each eye was stimulated individually by 3 consecutive flashes (7.7 cd.s/m^2^) at 1 flash/second (1 Hz). Two tracings were obtained per eye. The refractive power of each eye was measured with streak retinoscopy (Welch Allyn, Mississauga, Canada) without any pharmacologic manipulation of pupil size.

This study used a randomized crossover design. Each dog was randomly assigned to either the anesthesia or sedation arm of the study, and after a two-week washout period they received the opposite treatment (anesthesia or sedation). Within each arm of the study, all dogs received both flash and pattern stimulation and the stimulation received first was also randomized. Both eyes from each dog received the same stimulation, one eye at a time, resulting in a total of 26 eyes tested.

For the sedation protocol, a 20-gauge intravenous catheter was placed in the cephalic vein and each dog was given 5 μg/kg dexmedetomidine (Dexdomitor, Zoetis Canada, Kirkland QC**)** and 0.3 mg/kg butorphanol (Torbugesic, Zoetis Canada, Kirkland, QC**)** intravenously. None of the dogs required additional sedation during the procedure.

For the general anesthesia protocol, a 20-gauge intravenous catheter was placed in the cephalic vein. No premedication was given. General anesthesia was induced with propofol (Baxter Corporation, Mississauga ON) intravenously to effect (range 4.5–12 mg/kg; median 6 mg/kg). All dogs were intubated with a cuffed endotracheal tube. General anesthesia was maintained with sevoflurane (Sevorane, AbbVie Corporation, Saint-Laurent QC) in oxygen as required to maintain a surgical plane of anesthesia (end-tidal (expired) sevoflurane 1.1–4.1%). Oxygen flow was maintained at 10–30 ml/kg/min. An intravenous infusion of Normosol-R **(**Hospira, Montreal QC**)** was administered at a rate of 5 ml/kg/hr. A multichannel physiological monitor (Datex-Ohmeda CardiocapTM/5 GE Healthcare, Finland Oy, Helsinki, Finland) was used to monitor arterial oxygenation, heart rate, non-invasive blood pressure (systolic, mean, and diastolic), respiratory rate, tidal, end-tidal CO_2_, and expired sevoflurane concentration. Oxygen saturation was maintained above the minimal acceptable level of 95%. Systolic blood pressure was monitored indirectly using a pressure cuff, sphygmomanometer, and Doppler; systolic pressure was maintained above 80 mmHg. Respiration rate was maintained above 10 breaths per minute, and manual ventilation was used when needed. Body temperature was maintained between 37.8 –38.8 °C.

**Fig. 3:**
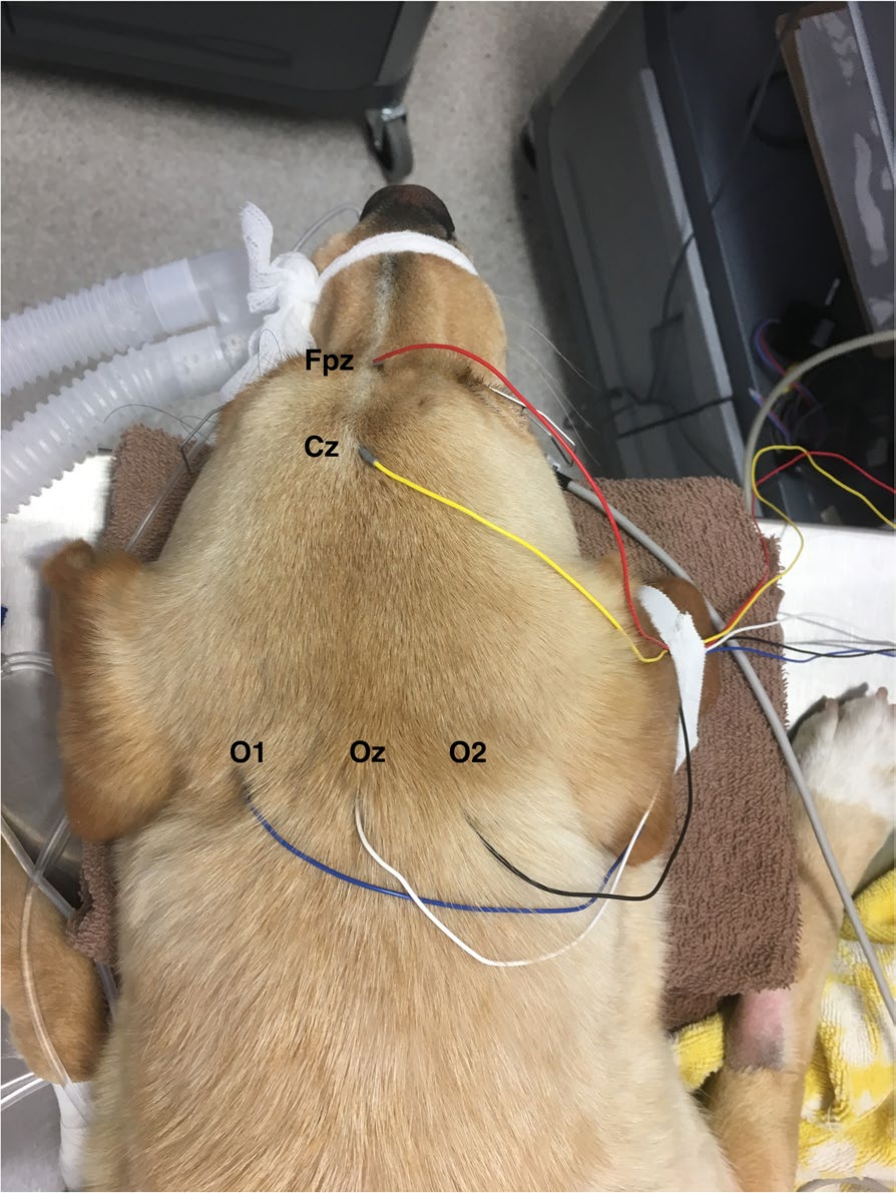
Electrode placement for VEP recording. The recording electrodes were placed over the inion, or dorsal occipital protuberance (O1, Oz, O2). The reference electrode was placed at the level of the forehead (Fpz). The ground electrode (Cz) was placed halfway between Oz and Fpz

The dogs were maintained under ambient lighting prior to data collection and recordings were obtained in the dark. As such, pharmacologic mydriasis was not performed. Twenty-seven gauge subcutaneous needle electrodes were positioned at Fpz (reference electrode), at O1, Oz and O2 (recording electrodes) and at Cz (ground electrode) (Fig. 3) as described previously for mesocephalic skulls [28]. Impedances were maintained at less than 4kΩ in all electrodes. One drop of Proparacaine 0.5% ophthalmic solution (Alcaine, Novartis Pharma Canada, Dorval QC**)** was applied to both eyes to provide topical anesthesia. Stay sutures were placed in the dorsal episclera of both eyes using 5–0 Monosof suture. Potentials were recorded one eye at a time. An eyelid speculum was used to retract the eyelids of the actively tested eye and gentle tension was applied to the sutures so that the pupil could be positioned centrally. The eye was kept hydrated using physiological saline flush between tests. The eye not being tested was covered with an opaque patch to avoid stimulation.

VEPs were recorded using a portable neurodiagnostic system (Sierra Sumitt, Cadwell, WA USA). The reference, recording and ground electrodes were connected to the positive, negative and ground inputs, respectively such that upward deflections were negative and downward deflections were positive in accordance with standard electrophysiology recording protocols [29]. The P-VEP signals at each of the 3 recording electrodes were averaged from 300 repetitions, and a total of 2 tests were done per eye. The high frequency filter was set to 100 Hz and the low frequency filter was set to 1 Hz. The testing distance from the eye to the stimulus display monitor (Dell, North York ON**)** was set at 22.9 cm [30]. The stimulus used was an alternating black and white checkerboard pattern with a stimulation rate of 3 reversals/second. The pattern size was 4 mm and the visual angle (check size) was 60 arc-min. The total pattern field size was 100.3 degrees. Mean luminosity was 34 cd/m^2^.

Patient setup, electrode placements and repetitions for F-VEP recording were the same as the pattern VEP procedure. Visual evoked potentials were recorded using a portable VEP system (Sierra Sumitt, Cadwell, WA USA). The signals at each of the 3 recording electrodes were averaged from 300 repetitions, and a total of 2 tests were done per eye. The high frequency filter was set to 100 Hz and the low frequency filter was set to 1 Hz. The photostimulator used to generate the stimulus was placed 20 cm away from the eye being tested. The flash stimulation rate was 1 flash/second (1 Hz) with an intensity of 2.5 cd/m^2^ and duration of 1 millisecond.

Latencies and amplitudes of observed peaks were measured according to previously published standards [2]. Latency was measured from the onset of the stimulus to the time of maximum positive or negative deflection [2]. Amplitude was measured from baseline to maximum deflection for the first peak and then from peak to peak in any recording with more than one peak [30]. For each individual animal, the results of both eyes were averaged together according to methods previously described [5]. The data were determined to be parametric using the Shapiro-Wilk test of normality, and the paired-samples T-test was used to compare data from each recording electrode between treatments. SPSS Statistics (IBM SPSS Statistics Version 24) was used for comparisons and a *P*-value of < 0.05 was the minimum acceptable level of significance.

## Supplementary Information

**Additional file 1.**

## Data Availability

All data generated or analysed during this study are included in this published article.
